# X-Rays

**Published:** 1906-08-11

**Authors:** 


### X-RAYS.

**Radiotherapy and Cancer.**—It has been frequently asserted that the treatment of cancer by *x*-rays has a dangerous side, apart from any liability to accident in the way of a "burn.". The fear of increasing the rapidity of growth or the liability to metastases has acted somewhat as a deterrent upon those who might otherwise have been inclined to resort to this method of treatment in inoperable cases. This fear Pusey [1] states to be groundless. Not only does he deny that there is increased danger of metastases, for in his experience he has never seen any case in which metastasis was induced or hastened by raying, but he goes so far as to say, on the contrary, that he has seen cases in which there was much reason to fear the occurrence of metastasis, show none after the *x*-rays had been used. From the outset there is a degeneration, not a stimulation; a distintegration, not a proliferation, of the youngest peripheral cells. Their vitality is lowered, and even where cells have been set free by the reactionary processes, they are in no condition to proliferate. On the whole, he considers that the rays have a restraining, and not a stimulating, action in carcinoma. In cancerous obstruction of the œsophagus Max Einhorn [2] continues to be satisfied with the results of his treatment with radium. He reports seven cases treated by means of a radium capsule passed in an œsophageal tube. In the case of a man aged 75, who had complete obstruction 15 inches from the teeth, improvement followed two and a half months' treatment on alternate days, so that he could swallow fluid or even semi-solid diet. Five months afterwards there was further improvement, and the patient had no pain. In five other cases there was improvement, swallowing being easier; while the seventh case showed no improvement.

**X-rays and Ringworm.**—Sabouraud's treatment of ringworn by the *x*-rays was merely put forward as a means of removing the hair in a rapid and painless manner. Statements have been made frequently that these rays have no bactericidal power in themselves, and in the laboratory this would seem to be the case. From Scherber's [3] researches, however, it would appear that the rays either in themselves are bactericidal, or more probably increase materially the power of the tissues to resist the invader. In sycosis, at any rate, the micro-organisms rapidly disappear, and the intense local reaction on the hair-sheaths and the papillæ favour this by impairing the chief seat of the invasion, the falling of the hairs enhancing the effect. Sichel,[4] however, although he admits that no other treatment of ringworm can claim such good results as even 50 per cent. of cures, seems disappointed that a method of such good promise is not productive of even better results. His experience has been in 102 cases of ringworm and parasitic alopecia. Of these only 35 were completed (30 tinea, 5 alopecia). Of the five alopecia cases four were cured, the fifth failing to attend after the hair had commenced to grow Out of 30 tinea cases 11 were cured; 10 were failures; 9 ceased attendance prematurely. He had one case of dermatitis among his latest patients, which seemed likely to lead to permanent baldness; this occurred in spite of great care in application, and makes an element of danger in treatment of 1 per cent. in his cases. On the other hand, the good results obtained in 50 per cent. of cases were completed in six months, as compared with a bare possibility of relief in six years by other methods. He thinks that the best results are obtainable in localised patches. He uses Sabouraud's pastilles, and gives an exposure of 20 to 25 minutes only unless the pastille has changed colour before this time. He enumerates the disadvantages as follows: (1) A specially-trained nurse is a necessity; (2) there is a possibility of an *x*-ray "burn," especially in blondes; (3) there is extreme difficulty in regulating the dosage of the rays; (4) after all the bother and expense the result may be a failure. The estimation of the colour change in the pastilles depends on several factors, of which personal judgment and the light in which the comparison with the standard is made are the chief. Other means at our disposal for estimating and controlling the rays comprise the following: The measurement of the spark gap; a milliammetre in the secondary circuit; the measurement of the interruptions of the primary circuit (McLeod), Benoist's radiometer, personal judgment in estimating the quality of phosphorescence in the tube (Schiff), length of time of exposure, the distance from the tube to the patient, the nearer the tube is to the patient the more superficial being the effect (Williams).[5]

**Renal Calculus and Skiagraphy.**—While it is conceded for the most part that there are certain fallacies in renal skiagraphy, such as, as Jacob [6] points out, fœcal concretions and calcareous glands and patches, Carl Beck [7] takes a very decided stand when he states that "a definite diagnosis in suspected lithiasis can be made in each and every instance; in other words, a renal calculus must invariably show, provided a calculus is present." Not only is this so, but the rays will reveal the size, shape, and number of such concretions, in the kidney, ureter, or bladder. The composition of the stone is of less importance than the necessity of bringing the calculous area as near to the plate as possible, and of keeping the area quiet. The tubular diaphragm is useful to obtain these two desiderata, while it allows the focal rays alone to pass through the aperture, cutting off the aberrant rays. The area permitted is 4 inches in diam.[8] in an adult, 3 inches in a child, and this area may prove too small, but its advantages are great. As the *modus operandi* he advises the following procedure: (1) Through evacuation of the bowels and the administration of a small dose of opium; (2) the head and shoulders should be elevated by pillows, while the chin touches the sternum; (3) the knees should be flexed and immobilised on sand bags; (4) the vacuum of the tube should be low. The criterion of a good plate is that the 11th and 12th ribs, the vertebræ, and the ilio-psoas muscle should show. If these do not appear, then the shadows are misleading, and the skiagram should not be used as a guide for operation. (5) If a plate show calculi, the compressor diaphragm should be used, elevating the shoulder of the exposed side more; the oblique direction permits of the diaphragm being pushed into the tissues further. (6) The bones should not show structural details. (7) Biliary stones should show if the tube be not too high. (8) A good vesical skiagram should show structural details of coccyx. (9) Turning patient sideways may allow the stone to move (vesical), and prove its mobility or the contrary on an oblique skiagram. (10) The *x*-rays are of more value than the cystoscope, as they will show embedded stones, and are less uncomfortable. (11) The renal region should be skiagraphed each time a vesical calculus is found. Beck states that he has found renal stone each time he has demonstrated a vesical one.

**Æsculin and Finsen Light in Lupus Vulgaris.**—Several attempts have been made to increase reaction of light by sensetising the tissues with fluorescent substances. After using 1 in 4,000 solution of erythrosin Graham [9] found much pain and even sloughing of the tissues ensue. He has used Æsculin with a Finsen-Reyn lamp with good results. Æsculin, a glucoside obtained from the horse chestnut, has the highest fluorescent properties. Morton has given one-grain doses without ill results.

Graham gave himself 5 minims of a 5 per cent. solution rendered alkaline by the addition of 3 per cent. sod. carbonate without bad result. The urine 15 minutes after showed slight fluorescence. After an hour the urine showed very distinct fluorescence, which increased markedly for four days after it was passed, *i.e.*, with greater alkalinity To prove that the tissues were rendered fluorescent he injected under the shaved skin of a guinea-pig 5 minims of a 5 per cent. solution. He then removed 3 square inches of skin with the subcutaneous and muscular tissue, and stretched these over a ring. He then passed the light from a Finsen-Reyn lamp through, and found that the whole of the skin and the subcutaneous tissue were very fluorescent, being 1 mil. thick, but in the muscle 3 mil. thick the fluorescence was not so evident. Only a small dose is necessary—1-5 min. of 5 per cent. solution—and it should be used within two days of being made. The reaction after light is usually over by the third day; after Æsculin it lasts four to seven days longer, and subsequent exposures continue to produce greater reactions for four to five days afterwards. No ill effects have followed 100 injections. The chief value Graham thinks to be in isolated, obstinate tubercular nodules, remaining after a preliminary course of Finsen treatment. It also softens thickened fibrous scars left after scraping for lupus, which render nodules often difficult of access.

[1] Annals of Surgery. Dec., 1905. [2] Berlin Klin. Woch, No. 44 A, 1905, Brit. Med. Jour., Jan. 6, 1906. [3] Edin. Jour., Jan., 1906. [4] Brit. Med. Jour., Feb. 3, 1906. [5] Med. Rec., Nov. 18, 1905. [6] Archives of Röntgen Ray, Mar., 1906. [7] Annals of Surgery, Dec., 1905. [8] Archives of Röntgen Ray, Feb., 1905. [9] Lancet, Dec. 16, 1905.

